# Microglia in morphine tolerance: cellular and molecular mechanisms and therapeutic potential

**DOI:** 10.3389/fphar.2024.1499799

**Published:** 2024-11-27

**Authors:** Xiangning Zhang, Tingting Jin, Haixia Wang, Shuai Han, Yongxin Liang

**Affiliations:** ^1^ Department of Anesthesiology, Women and Children’s Hospital, Peking University People’s Hospital, Qingdao University, Qingdao, Shandong, China; ^2^ Clinical Medical College, Qingdao University, Qingdao, Shandong, China; ^3^ Department of Anesthesiology, Northern Jiangsu People’s Hospital Affiliated to Yangzhou University, Yangzhou, Jiangsu, China; ^4^ Clinical Medical College, Yangzhou University, Yangzhou, Jiangsu, China

**Keywords:** morphine tolerance, microglia, signal transduction, opioids, tolerance mechanisms

## Abstract

Morphine has a crucial role in treating both moderate to severe pain and chronic pain. However, prolonged administration of morphine can lead to tolerance of analgesia, resulting in increased doses and poor treatment of pain. Many patients, such as those with terminal cancer, require high doses of morphine for long periods. Addressing morphine tolerance can help this group of patients to escape pain, and the mechanisms behind this need to be investigated. Microglia are the key cells involved in morphine tolerance and chronic morphine administration leads to microglia activation, which in turn leads to activation of internal microglia signalling pathways and protein transcription, ultimately leading to the release of inflammatory factors. Inhibiting the activation of microglia internal signalling pathways can reduce morphine tolerance. However, the exact mechanism of how morphine acts on microglia and ultimately leads to tolerance is unknown. This article discusses the mechanisms of morphine induced microglia activation, reviews the signalling pathways within microglia and the associated therapeutic targets and possible drugs, and provides possible directions for clinical prevention or retardation of morphine induced analgesic tolerance.

## 1 Introduction

Opioid analgesics are irreplaceable drugs for the clinical treatment of chronic pain, cancer pain, intraoperative analgesia and postoperative analgesia. However, long-term use of opioids can trigger opioid tolerance, which in turn leads to increased doses, leading to more serious side effects such as respiratory depression, sedation, constipation, dependence, and addiction (Ing Lorenzini et al., 2022). The search for mechanisms for the development of tolerance and clinically alternative dosing regimens has therefore become particularly urgent. Morphine is the classic opioid. Numerous studies have shown that morphine activates the neuroinflammatory response, activating glial cells and promoting the release of inflammatory cytokines such as interleukin (IL)-1β, IL-6, IL-18, and tumour necrosis factor (TNF)-α (Cai et al., 2016; Pan et al., 2016; Wang et al., 2009; Zhang et al., 2017; Zhou et al., 2010). Microglia, differentiated from haematopoietic stem cells, are the outpost cells of infection and injury. They account for approximately 5% of human neuroglia and 5%–20% of all rodent glial cells (Saijo and Glass, 2011). Microglia are key cells involved in morphine tolerance, and morphine activates internal microglia signalling pathways and increases the expression of inflammatory factors. Inhibitors of microglia can alleviate morphine tolerance (Cui et al., 2008), however, the molecular mechanisms involved are not yet clear.

In this paper, we explore the signalling pathways involved in microglia associated with morphine tolerance based on existing research, and summarise the basic research available, including possible combination regimens and potential targets, in the hope of providing new ideas for clinical pain management and future research on morphine tolerance.

## 2 Microglia activation and morphine tolerance

Microglia are derived from mesodermal bone marrow haematopoietic stem cells, which are specialised “macrophages” of the central nervous system (CNS) (Saijo and Glass, 2011). Although it was realised early on that there were significant differences in the analgesic effects of morphine across sexes and in the tolerance of long-term morphine administration (Craft et al., 1999), the mechanisms underlying such differences remained unclear. It has been shown that minocycline, a microglia inhibitor, enhances the analgesic effect of morphine in male rats but does not affect the outcome in female rats (Posillico et al., 2015), suggesting that there may be a sex difference in the activation of microglia in morphine analgesia, but the influence of the different effects of minocycline in different sexes cannot be ruled out. Reiss et al. (2022) found that knockout of μ-opioid receptors in microglia did not result in sex differences in tolerance, but did result in sex differences in nociceptive hypersensitivity (OIH): OIH disappeared in knockout male mice but persisted in females. A recent study showed gender differences in LPS-induced activation of microglia in rats (Nikodemova et al., 2024), and it is not clear whether the same difference exists for activation of microglia by morphine. It may be precisely because morphine has better analgesic effect and tolerance in males (Craft et al., 1999), which most of the previous studies on morphine tolerance have selected only male rats/mice as the study subjects, and we believe that there is value in having a study on morphine tolerance and gender dimorphism in microglia.

**FIGURE 1:**
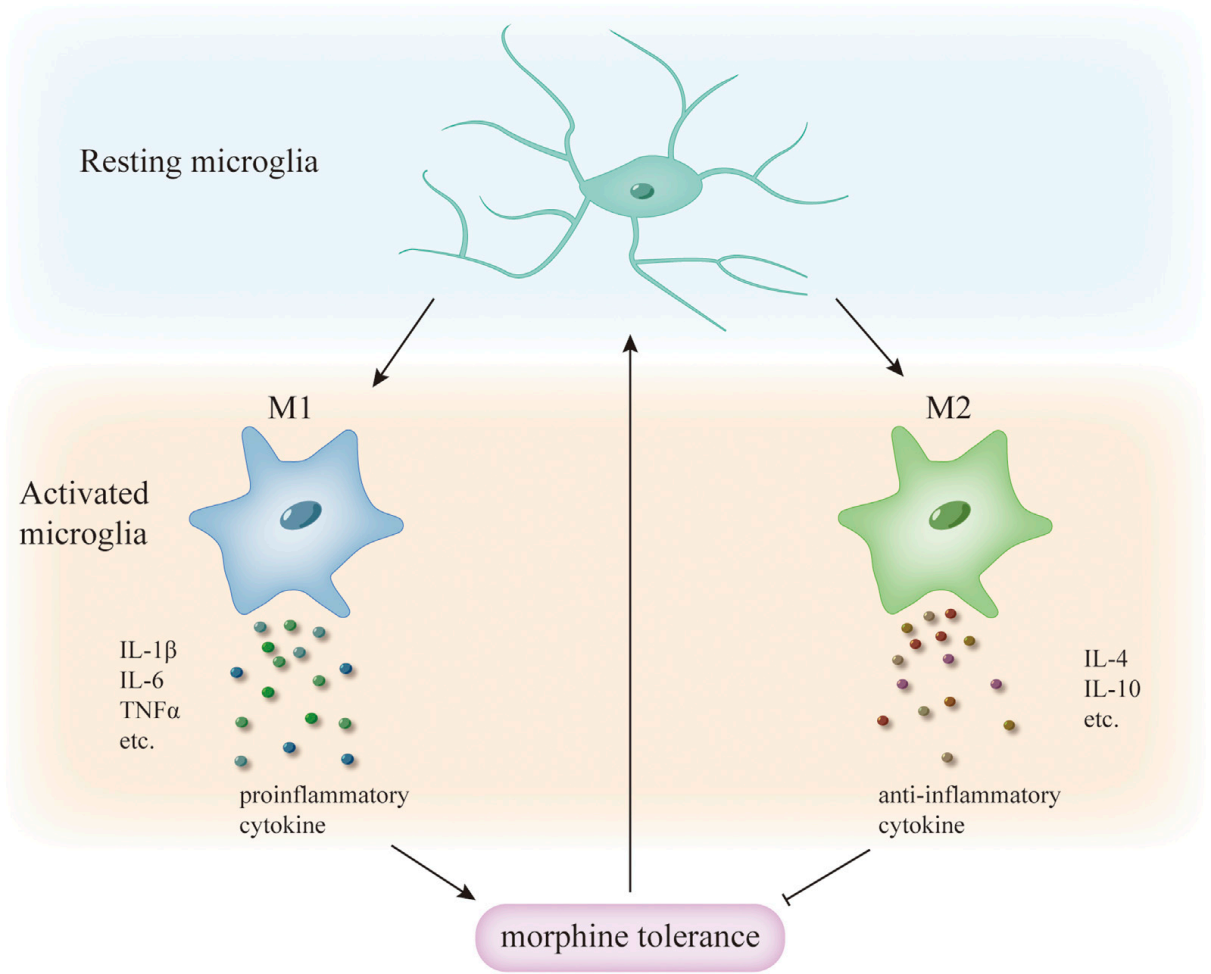
Different phenotypes of microglia activation and morphine tolerance. During morphine tolerance, microglia transform from a resting to an activated phenotype, with different activation phenotypes releasing different cytokines; M1 microglia release pro-inflammatory cytokines, whereas M2 microglia release anti-inflammatory cytokines, which enhance tolerance, and anti-inflammatory cytokines, which delay tolerance. Therefore, inducing microglia to transform from M1 to the M2 phenotype may be able to attenuate morphine tolerance.

Microglia can respond rapidly to stimuli of infection and injury, rapidly changing their morphology to an activated state of “amoeba”. They are transformed into different forms by different stimuli, the more common being M1 “classical activation” and M2 “selective activation”, with M1 releasing large amounts of proinflammatory cytokines and M2 releasing anti-inflammatory cytokines (Xu et al., 2020). Different microglia predominate at different times after injury (Li et al., 2022), whereas during morphine tolerance, microglia are activated and transformed towards M1 and M2 phenotypes, with M1 releasing large amounts of inflammatory factors that promote the development of morphine tolerance (Tu et al., 2021), in contrast, the anti-inflammatory effect of M2 microglia inhibits the development of morphine tolerance to some extent (Jokinen et al., 2018) (Figure 1). In fact, M2 microglia were subdivided into three different subtypes in the study, and there was crosstalk between the different subtypes of microglia (Li et al., 2022), whereas the roles played by the different subtypes of M2 microglia in morphine tolerance are still not fully understood, and further studies are needed in the future. Thus, in conclusion, the above results suggest that promoting the transformation of M1-type microglia to M2-type may be able to inhibit the development of morphine tolerance.

Previous studies have suggested that morphine activates microglia and initiates downstream signalling by acting on toll-like receptor 4 (TLR4) (Eidson and Murphy, 2013), calcitonin gene-related peptide (CGRP) (Wang et al., 2010a; 2010b; 2009), P2X7Rs (Chen et al., 2012; Zhou et al., 2010) and μ-opioid receptors (MOR) (Merighi et al., 2013; 2012). However, the controversial point remains whether microglia can express MOR (Cataldo et al., 2019; Corder et al., 2017; Kao et al., 2012; Maduna et al., 2019). Opioids such as morphine exerts pharmacological effects and side effects by acting directly on MOR (Günther et al., 2018). Some studies have suggested that microglia do not express MOR (Corder et al., 2017; Kao et al., 2012). Corder et al. (2017) demonstrated by gene sequencing, selective knockdown of MOR and the use of peripheral antagonists of MOR that microglia do not express MOR, but rather that MOR on peripheral primary afferent injury receptors is involved in the development of morphine tolerance. In contrast, other studies have suggested that microglia express MOR (Cataldo et al., 2019; Maduna et al., 2019), as Maduna et al. (2019). demonstrated that microglia express MOR and its associated proteins through analysis of the microglia gene transcriptome from humans and rodents. Similarly, Reiss et al. demonstrated this by comparing MOR expression levels in microglia from MOR knockout (cKO) and control groups of mice (Reiss et al., 2022). We suggest that the differences in the results of these studies may be related to the different phenotypes of microglia and may also result from the different experimental methods and the different experimental environments.

In recent years, several new targets have been identified, such as the platelet-derived growth factor receptor β subunit (PDGFRβ), and continuous intrathecal injection of the PDGFRβ inhibitor imatinib prior to morphine administration attenuates morphine tolerance and reduces the expression of Iba1, a marker of microglia activation, in the rat spinal cord (Li et al., 2020). In addition, the results of another *in vivo* experiment showed that the microglia marker CD11b colocalization with epidermal growth factor receptor (EGFR) in the rat spinal cord, and that intrathecal injection of the EGFR antagonist AG1478 reduced the expression of CD11b and increased the analgesic effect of morphine (Yang et al., 2021b). Another study based on a mouse model of neuropathic pain (NCP) found that morphine was able to activate the aplin receptor (APLNR) to palmitoylate it and, through its downstream ERK1/2 signalling, to activate microglia, and that inhibition of the APLNR was able to reverse the morphine-induced increase in Iba-1 expression (Fan et al., 2024). It has also been found that microglia activation associated with morphine tolerance occurs at the spinal cord level (Jokinen et al., 2018). Chronic morphine administration upregulated the expression of the microglial cell marker Iba1 as well as CD11b in the spinal cord (Horvath and DeLeo, 2009; Zhou et al., 2010), which are barely expressed in the resting state of microglia. Activation of microglia in the spinal cord or in the cerebral cortex leads to the release of different inflammatory or anti-inflammatory factors (Nikodemova et al., 2024). Therefore, if microglia activation in the spinal cord can be selectively inhibited, it may be possible to reduce morphine tolerance.

## 3 Signaling pathways in microglia and morphine tolerance

### 3.1 TLR signalling pathway

Toll-like receptors (TLRs) belong to the TIR receptor superfamily, which consists of two subgroups: Toll-like receptors and interleukin (IL)-1 receptors (Liew et al., 2005). The different TLRs are distributed in different cells and they play an influential role in immunity (McGuire and Arthur, 2015). Among them, TLR4 serves as an important signal involved in morphine tolerance (Liang et al., 2016; Wang et al., 2021; Zhang et al., 2017). It has been previously demonstrated that TLR4, mainly in microglia, is involved in the development of morphine tolerance and that the use of TLR4 antagonists can reduce morphine tolerance in a dose-dependent manner (Eidson and Murphy, 2013; Wang et al., 2021). Some studies have shown that morphine is able to bind specifically to the LPS-binding pocket of myeloid differentiation protein 2 (MD-2), a TLR4 accessory protein, and induces an inflammatory response in microglia via the TLR4/MD-2 complex (Hutchinson et al., 2010; Wang et al., 2012). This may be the mode of action of TLR4 in morphine tolerance. Currently known upstream signals of TLR4 in morphine tolerance include the transcription factor TCF7L2, the cannabinoid receptor CB2 and the high mobility group box-1 (HMGB1) (Chen et al., 2021; Lin et al., 2023; Ma et al., 2021). TCF7L2 is an important transcription factor that is upregulated in models of neuropathic pain (Xu Z. et al., 2015; Zheng et al., 2019). Chronic morphine administration increases the expression of TCF7L2, which is able to transcriptionally regulate the expression of TLR4 receptors and influence TLR4 downstream signalling (Chen et al., 2021). The low-dose cannabinoid receptor CB2 agonist AM1241 was able to regulate TLR4 mRNA expression in morphine-tolerant mice, which in turn regulated TLR4 and its downstream p38 MAPK signalling pathway (Ma et al., 2021). HMGB1 is a heat shock protein, and both in vivo and *in vitro* experiments, morphine increased the expression of HMGB1, while *in vitro* experiments demonstrated that HMGB1 released from neurons activated TLR4 on microglia and activated its downstream signalling (Lin et al., 2023). Next, TLR4 activation activates microglia and initiates downstream signalling pathways, such as NF-κB, MAPK and NLRP3 (Lehnardt et al., 2003; Lin et al., 2023; Olson and Miller, 2004). Studies have demonstrated that morphine is able to increase the phosphorylation of p65 and p38 by activating TLR4 signalling (Chen et al., 2021; Pan et al., 2016). Moreover, activation of TLR4 in microglia also promotes phosphorylation of TGF-β-activated kinase 1 (TAK1), and inhibition of increased phosphorylation of TAK1 attenuates morphine tolerance and does not affect TLR4 expression (Wang et al., 2021), suggesting that TLR4 and its downstream signal TAK1 are involved in morphine tolerance. TAK1 is a MAPK kinase kinase family, the most common upstream kinase of MAPK (Chen et al., 2015), and one of the upstream signals of NF-κB (Liew et al., 2005). Therefore, in morphine tolerance, TLR4 may regulate p38 MAPK and NF-κB signalling indirectly by activating TAK1. Furthermore, TLR4 in microglia is also able to regulate receptor transporter protein 4 (RTP4) expression in the hypothalamus, and knockdown of RTP4 attenuates tolerance (Fujita et al., 2022).

Recently, another study showed that TLR2 expression is increased during morphine tolerance and that inhibition of microglia using minocycline was able to reduce TLR2 expression and attenuate morphine tolerance (Peng et al., 2023). However, due to the limitations of this study, the cellular localisation of TLR2 is unknown and whether it is TLR2 on microglia that is involved in this process remains unclear and requires further investigation. But this result suggests that TLRs in addition to TLR4 signalling, TLR2 is also involved in morphine tolerance and is associated with microglia activation.

Briefly, among the Toll-like receptor family, TLR4 in microglia plays an important role in the development of morphine tolerance, which is an essential signal for microglia activation as well as for triggering the cascade reaction (Eidson and Murphy, 2013; Wang et al., 2021), while TLR2 is in turn involved in the signalling circuit of morphine tolerance and microglia activation (Peng et al., 2023), and morphine tolerance in rats with selective knockout of TLR2/4 attenuated (Derangula et al., 2022), thus TLRs are expected to be new targets for delaying morphine tolerance and pain diagnosis and treatment.

### 3.2 p38 MAPK and NF-κB signalling pathways

Mitogen-activated protein kinases (MAPK) are a group of serine-threonine protein kinases that can be activated by a variety of stimuli, such as cytokines, growth factors, neurotransmitters, and hormones (Widmann et al., 1999). P38 MAPK is one of five families of mammalian MAPKs; the other families include extracellular signal-regulated kinases 1/2 (ERK1/2), c-Jun N-terminal Kinase (JNK), ERK3/4, and ERK5 (Widmann et al., 1999). Among these, the p38 MAPK pathway in spinal microglia is involved in the development of morphine tolerance (Cui et al., 2006; Liu W. et al., 2006), and pharmacological blockade of p38 MAPK attenuates morphine tolerance (Gong et al., 2021). In addition to TLR4, there are many other signals upstream of p38 MAPK during the development of morphine tolerance: PDGFRβ mediates cellular autophagy via p38 (Jia et al., 2021), calcitonin gene-related peptide (CGRP) (Wang et al., 2009) and P2X7 (Zhou et al., 2010) all regulate the activation of p38 MAPK in microglia. Interestingly, Cui et al. found that the administration of minocycline to rats starting on day 8 after morphine administration reduced the activation of p38 in spinal microglia but did not reverse the established morphine tolerance, suggesting that the p38 MAPK pathway may be more involved in the development than in the maintenance of morphine tolerance (Cui et al., 2008). At the same time, it was shown that gene silencing of E3 ubiquitin ligase Pellino1 (Peli1) in the spinal cord delays the development of morphine tolerance but does not reverse the tolerance that has already been established, a suggestion supported by the fact that Peli1 is involved in morphine tolerance via the MAPK pathway (Wang L. et al., 2020). In addition, morphine activates Nuclear factor-kappaB (NF-κB), an important transcription factor responsible for the transcription of inflammatory factors in neurons and microglia, and translocates it from the cytoplasm to the nucleus (Chen et al., 2006; Pan et al., 2016). NF-κB is involved in several intracellular signalling pathways, such as the cAMP/protein kinase A (PKA)/cAMP reaction (CREB) pathway, the PI3K/Akt/IκB kinase complex inhibitor (IKK) pathway, and the TLRs pathway (McGuire and Arthur, 2015; Ye, 2001).

Calcitonin gene-related peptide (CGRP) is a neuropeptide widely distributed in the peripheral and central nervous system, including the dorsal root ganglion (DRG) and its primary afferent terminals in the spinal cord, and is involved in the regulation of injury perception (Rosenfeld et al., 1984; Tomas et al., 1992). It has been shown in numerous studies that CGRP signalling activates p38 and NF-κB signalling in microglia and is involved in morphine tolerance, while the mu-opioid receptor (MOR) is involved in the regulation of CGRP as an upstream signal (Jokinen et al., 2018; Wang et al., 2010a; 2010b; 2009; Zadina et al., 2016). Furthermore, elevated expression of the neuronal activation marker c-fos has been suggested as an indicator of morphine tolerance in recent studies (Pan et al., 2016; Wang L. et al., 2020), and application of exogenous CGRP also increased c-fos expression in microglia (Wang et al., 2010b). Adrenomedullin (AM) belongs to the calcitonin gene-related peptide (CGRP) family. AM is involved in the activation of microglia in the morphine tolerance process and affects the expression of inflammatory factors (Zeng et al., 2014).

Activation of p38 MAPK and NF-κB signalling by upstream signals, including TLR4, increases the expression of inflammatory factors such as IL-1, IL-6, IL-18 and TNF-α (Cai et al., 2016; He et al., 2014; Wang L. et al., 2020), while some inflammatory factors such as IL-1β and TNF-α, in turn, activate p38 and NF-κB signalling in microglia through their correlated receptors (Oeckinghaus et al., 2011; Skaug et al., 2009), which may accelerate the establishment of tolerance in the early stages of morphine tolerance (Lin et al., 2015). Besides, NLRP3 inflammasome are another important signal downstream (Cai et al., 2016; Chen et al., 2021; Wang et al., 2021; Zhang et al., 2017). Knockdown of NLRP3 reduces microglial activation, attenuates morphine tolerance and affects pain thresholds in mice (Liu et al., 2020; Wang H. et al., 2020). NLRP3 inflammasome, which consists of the apoptosis-associated particulate protein ASC, caspase-1 and NLRP3, is able to activate caspase-1 and promote the conversion of pro-IL-1β and pro-IL-18 to IL-1β and IL-18 (O’Neill, 2008). Whereas Ac-YVAD-cmk (YVAD), a selective irreversible inhibitor of caspase-1, delays morphine tolerance (Hutchinson et al., 2008), it reduces the expression of IL-1β in microglia *in vitro* (Liang et al., 2019). Phosphorylation of TAK1, the upstream signal of p38, can increase NLRP3 expression, while knockdown of NLRP3 does not affect TAK1 expression (Wang et al., 2021). The current study shows that upstream signals of NLRP3 inflammasome include P2X7R (Cai et al., 2016; Wang H. et al., 2020) and ROS (Juliana et al., 2012) signals in addition to p38 MAPK/NF-κB, which are involved in NLRP3 inflammasome activation during the onset of morphine tolerance. In addition, melatonin is able to alleviate morphine tolerance by reducing levels of NLRP3, TLR2 (Peng et al., 2023) and ROS(Chen et al., 2020; Liu et al., 2020). Zingerone delayed morphine tolerance by inhibiting NLRP3 inflammasome and oxidative stress, and it was experimentally demonstrated that zingerone was able to reduce morphine-induced protein of IL1β, NLRP3, caspase-1, and ASC expression increases (Molavinia et al., 2024). Taken together, these studies illustrate the potential role of NLRP3 signalling in the progression of morphine tolerance.

A recent study demonstrated that metformin attenuated morphine tolerance by inhibiting the activity of the TLR4/p38 MAPK/NF-κB pathway. Morphine was able to induce translocation of p65 NF-κB from the cytoplasm to the nucleus and enhance phosphorylation of p38 MAPK, and TLR4 expression, but both were inhibited by metformin (Pan et al., 2016). Procyanidins (an NLRP3 inhibitor) inhibited morphine-induced NF-κB translocation and increased phosphorylation of p38 MAPK (Cai et al., 2016).

In conclusion, p38 MAPK/NF-κB signalling in microglia is involved in the development of morphine tolerance, where positive inflammatory factor-receptor-transcription factor feedback signalling may be a key factor influencing the establishment of morphine tolerance. Drugs targeting this signalling pathway and its associated regulatory signals may be able to alleviate morphine tolerance.

### 3.3 P2X4 and P2X7 signaling pathways

P2X4 is involved in the activation and migration of microglia and the formation of morphine tolerance, and inhibitors of the P2X4 receptor (P2X4R) can reduce morphine tolerance (Horvath et al., 2010; Zeng et al., 2021). Morphine activates P2X4Rs in microglia via ATP, which in turn causes the release of brain-derived neurotrophic factor (BDNF) (Ferrini et al., 2013). In turn, BDNF is involved in the regulation of vesicular glutamate transporter protein (VGluT2) expression during morphine tolerance, causing an increase in glutamate release (He et al., 2022), which may contribute to P2X7 receptor activation (see below for the specific mechanism). In addition, μ opioid receptors may be involved in the upregulation of P2X4Rs in microglia (Ferrini et al., 2013). In contrast, in another study, the inhibitor antisense oligonucleotide (asODN), which inhibits the function and expression of P2X4R, suppressed the morphine-induced increase in mu-opioid receptor protein expression (Horvath et al., 2010). It can therefore be hypothesized that μ receptors and P2X4 receptors promote one another’s activation during morphine tolerance formation, facilitating the upregulation of the other’s expression on the cell membrane surface.

The purinergic P2X7 receptor (P2X7R) is involved in the acute analgesia of morphine (Zeng et al., 2021). It has been shown in many studies that P2X7 is involved in the development of morphine tolerance and that morphine tolerance can be attenuated by inhibiting P2X7R activity (Chen et al., 2012; Wang H. et al., 2020; Zadina et al., 2016; Zhou et al., 2010). Morphine tolerance leads to increased glutamate concentrations in rat cerebrospinal fluid (Wen et al., 2004), which in turn may lead to excessive ATP release from spinal glial cells in an AMPA receptor-mediated calcium-dependent manner (Liu G. J. et al., 2006; Zhou et al., 2010), which ultimately activates spinal P2X7R via ATP. Additionally, activation of P2X7R will in turn cause ATP and glutamate release, and this positive feedback may contribute to morphine tolerance in sustained activation of P2X7R (Ye et al., 2003). In turn, glutamate transporter proteins are critical for the analgesic effects of morphine (Zhou et al., 2010). In an *in vitro* experiment based on primary microglia from rat spinal cord, experiments suggest that Src kinase may be involved in morphine-induced activation of P2X7R (Leduc-Pessah et al., 2017). The site of action may be the P2X7R located at the intracellular C-terminal Y_382–384_ site, which contains three tyrosine residues (Leduc-Pessah et al., 2017). P2X7R activation and Ca^2+^ influx promote activation of the p38 MAPK, IL-1β or IL-18 pathways in microglia, followed by IL-18 activation of IL-18R, leading to activation of astrocytes and triggering a series of inflammatory responses (Chen et al., 2012; Wang H. et al., 2020). In addition, inhibition of P2X7R in the spinal cord reduces the activation of NLRP3 inflammasome during morphine tolerance (Wang H. et al., 2020).

In conclusion, P2X4 and P2X7 are involved in the establishment of morphine tolerance and may have a reciprocal regulatory role, however, the mechanisms involved need to be further investigated. Notably, the regulatory role of P2X7 signalling on the p38 MAPK/NLRP3 inflammasome signalling pathway also plays a role in morphine tolerance. This suggests that the establishment and maintenance of morphine tolerance is a complex process in which multiple signalling pathways are involved in regulating and promoting each other. Therapeutic agents targeting P2X4R or P2X7R could be a new approach to alleviate morphine tolerance. In turn, the series of signalling pathways they trigger may become new therapeutic targets for reducing morphine tolerance.

### 3.4 PTK family signalling pathways

Protein tyrosine kinases (PTKs) are a large family of receptor-and non-receptor-type tyrosine kinases. Epidermal growth factor receptor (EGFR) and platelet-derived growth factor receptor (PDGFR) are two very common members of the receptor-type tyrosine kinase family (Dr et al., 2000; Hubbard and Till, 2000). Yang et al. demonstrated through *in vivo* experiments based on rats and *in vitro* experiments based on BV2 cell lines that morphine activates EGFR and its downstream ERK signalling and activates microglia, and that EGFR inhibitors reduce morphine tolerance and inhibit the expression of inflammatory factors (Yang et al., 2021b; Yang et al., 2021a). In another study, the EGFR antagonist gefitinib was able to prevent morphine tolerance but had no analgesic effect by itself (Puig et al., 2020). Chronic administration of morphine increases the phosphorylation of PDGFRβ and p38 MAPK in microglia (Jia et al., 2021), while the study found that PDGFRβ is involved in microglia activation and that JNK may be an upstream signal in this pathway (Li et al., 2020). Moreover, there is a reciprocal regulation of EGFR and PDGFRβ in the regulation of mechanical abnormal pain (Puig et al., 2020), and this mechanism may also be involved in morphine tolerance. tropomycin receptor kinase B (TrkB) is a receptor for BDNF, which belongs to the nerve growth factor receptor family, a subfamily of the receptor-type tyrosine kinase family (Ségaliny et al., 2015). The involvement of TrkB in morphine tolerance is controversial; Ferrini et al. found that antibodies to TrkB did not reduce morphine tolerance (Ferrini et al., 2013), whereas He et al. successfully reduced morphine tolerance using the tyrosine kinase inhibitor K252a (He et al., 2022). The same morphine concentrations were used in both studies, but it is possible that different blocking mechanisms of TrkB/BDNF signalling or different rodents (rat/mouse) contributed to the different results. Notably, in Ferrini et al.’s experiments, TrkB antibodies were able to inhibit morphine-induced nociceptive hypersensitivity (OIH) (Ferrini et al., 2013), suggesting a different mechanism for the development of OIH and tolerance.

**FIGURE 2:**
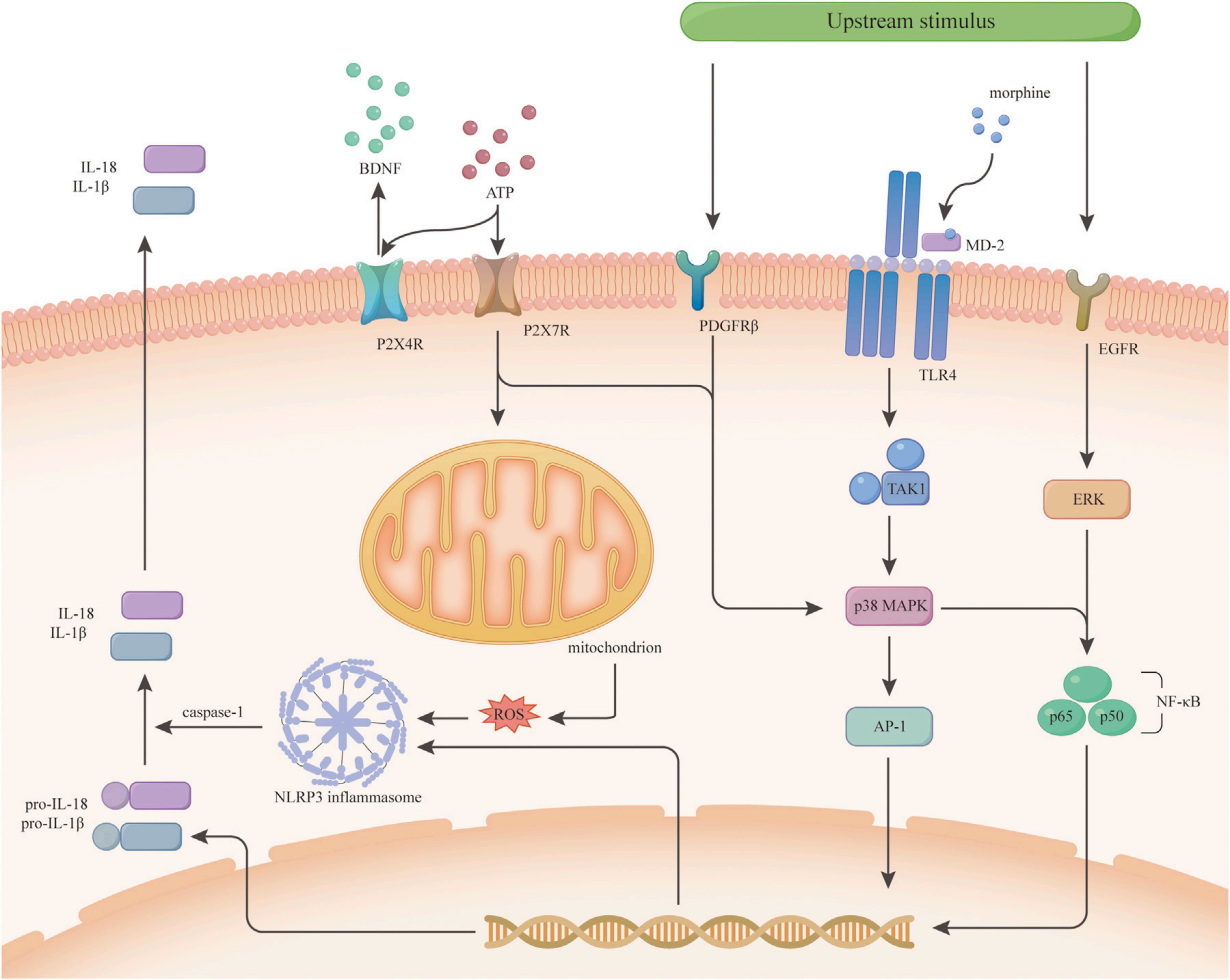
Morphine tolerance-related signaling pathways inside microglia. This figure is a speculation of some of the signaling pathways inside microglia based on current research. P2X4R and P2X7R activate different downstream signals upon ATP stimulation. P2X4R releases BDNF, while P2X7R activates mitochondria and p38 MAPK to increase inflammatory cytokine release. Some receptors on microglia such as PDGFRβ, TLR4, and EGFR activate different signals downstream after some upstream stimulus. The TLR4/p38 MAPK/NF-κB signaling pathway was demonstrated early on in morphine tolerance, and p38 MAPK in this pathway is crosstalk, which has various upstream signals, not only under the regulation of TLR4. Both PDGFRβ and EGFR activate NF-κB through the MAPKs family and initiate transcription to increase the expression of inflammatory cytokines.

In conclusion, EGFR, PDGFRβ and TrkB of the PTK family are important players in microglia activation and morphine tolerance (Puig et al., 2020; Puig and Gutstein, 2023; Yang et al., 2021b; Yang et al., 2021a). Also, MAPKs signalling is a key crossroads, but the exact mechanisms need to be further investigated and whether other protein tyrosine kinases are involved in morphine tolerance remains to be determined. EGFR and PDGFRβ are expected to be new targets for improving morphine analgesia (Figure 2).

### 3.5 Other pathways

Morphine activates PKCε and activates the downstream Akt/ERK signalling pathway, resulting in increased release of nitric oxide and inflammatory factors Morphine increased CXCL10 expression in microglia, which acts on CXCR3 on neurons, and inhibition of CXCR3 was able to reduce tolerance (Wang et al., 2017). This suggests that this signal is involved in the interaction between microglia and neurons during tolerance, however, the exact mechanism remains unclear. Microparticles (MPs) are extracellular vesicles, and once synthesised in cells, inflammatory factors need to be released via vesicle encapsulation. Recent studies have shown that blood-derived MPs are involved in the activation of microglia and the formation of morphine tolerance and have confirmed that inflammatory factors such as IL-1β are encapsulated in MPs (Ruhela et al., 2020). The vesicular glutamate transporter protein VGluT2 is involved in microglia activation and the BDNF/TrkB pathway is an upstream pathway for its expression regulation (He et al., 2022). As previously mentioned, P2X4Rs are able to elicit the release of BDNF, and it is speculated that P2X4 may also be involved in the regulation of VGluT2 expression. Knockdown of VGluT2 can inhibit the development of morphine tolerance (He et al., 2022).

## 4 Treatment strategy

In a previous study, the microglia inhibitor minocycline was able to reduce morphine tolerance by inhibiting p38 MAPK in microglia (Cui et al., 2008). Many of the signalling pathways mentioned above could be used as new targets to improve morphine tolerance. In addition, several drug combination regimens have been shown to serve as new strategies for clinical opioid analgesia.

### 4.1 Drugs

Candesartan is a commonly used angiotensin II receptor type 1 blocker that inhibits morphine-induced activation of microglia, thereby reducing morphine tolerance (Zhao et al., 2022). Candesartan increases the expression of peroxisome proliferator-activated receptor (PPAR)γ and 5′-adenosine monophosphate-activated protein kinase (AMPK) in morphine-induced BV2 cells and reduces the expression of inflammatory factors in morphine-induced BV2 cells by activating the PPARγ/AMPK signalling pathway (Zhao et al., 2022). While metformin is an AMPK agonist, it has been shown in recent studies to reduce morphine tolerance without affecting basal analgesia (Pan et al., 2016; Shirooie et al., 2020; Wan et al., 2022). Metformin inhibits morphine-induced microglia activation by activating AMPK (Pan et al., 2016), it increases the expression of suppressor of cytokine signalling pathway 3 (SOCS3) in microglia, and silencing SOCS3 significantly inhibits the anti-inflammatory effects of metformin (Wan et al., 2022). Glibenclamide is a K_ATP_ pathway inhibitor and also a commonly used drug in diabetic patients. It has been shown in recent studies to attenuate morphine tolerance and inhibit morphine-induced microglial activation. Also, glibenclamide inhibited morphine-induced activation of NLRP3 inflammasome (Qu et al., 2017). We summarise some of the drugs that have targeted microglia activation-related targets in recent animal studies (Table 1), which may provide some new directions for the clinical use of opioids and subsequent studies on microglia and opioid tolerance.

**TABLE 1:** Drugs that target microglia to delay morphine tolerance.

Therapeutic drugs	Administration	Targets	Ref.
Teneligliptin	*in vivo*: rats, morphine (15 μg/μL, h, i.t.) and Teneligliptin (2 μg/μL, h, i.t), 7 d	Nrf2, HO-1	Kuthati et al. (2023)
Corilagin	*in vitro*: BV2 cells, Corilagin (0.1, 1 or 10 μM)18 h, then Corilagin (0.1, 1 or 10 μM) &morphine (200 μM) 6 h	TLR2	Guan et al. (2023)
Melatonin	*in vivo*: rats, morphine (10 μg/5 μL, i.t., b.i.d.), 7 d, melatonin (60 μg/5 μL, i.t.), 30 min before morphine administration (Peng et al., 2023)	TLR2/NLRP3	Chen et al. (2020), Lin et al. (2016), Liu et al. (2020), Peng et al. (2023)
	*in vivo*: mice, morphine (10 mg/kg, s.c., q.d.)7/14/21 d, melatonin (0.5 mg/kg, i.p.), 30 min before morphine administration, 7/14/21 d *in vitro*: BV2 cells, melatonin (ethanol solution, 200 μM) 30 min, then morphine (200 μM, 6 h) or LPS(1 μg/mL, 6 h) (Liu et al., 2020).	NLRP3	
	*in vivo*: rats, morphine (15 μg/h, i.t.) and melatonin (3, 6 or 12 g/h, i.t.), 5 d (Chen et al., 2020)	Antioxidative Enzymes	
	*in vivo*: rats, morphine (15 μg/h, s.c.), 7 d, then 3 hours later melatonin (10 μg/h, s.c., 30 min) (Lin et al., 2016)	HSP27	
Bulleyaconitine A (BAA)	*in vivo*: rats, morphine (10 mg/kg, s.c., b.i.d.), 10 d, BAA (0.4 mg/kg, i.g.), 30 min before morphine administration, 10 d (Mai et al., 2020)	PKCγ	Li et al. (2016), Mai et al. (2020)
	*in vivo*: rats, morphine (20 mg i.t., b.i.d.) and BAA (300 ng, i.t., bid), 7 d (Li et al., 2016)	dynorphin A	
Glucosamine	*in vivo*: mice, morphine (20 mg/kg, s.c., b.i.d.), 9 d, glucosamine(500, 1,000 and 2000 mg/kg, i.g.) 30 min before morphine administration, 9 d	iNOS, TLR4	Basiri et al. (2019)
Extracts of Hericium erinaceus (EHE)	*in vitro*: BV2 cells, EHE (1 ng-1 μg/mL) 30 min, then morphine (10–100 μM, 2 h)	HDAC 6/HSP90	Yeh et al. (2019)
Lidocaine	*in vivo*: mice, morphine (10 μg/10 μL, i.t., q.d.)and lidocaine (100, 200 and 400 μg/10 μL, i.t., q.d.), 7 d; *in vitro*: BV2 cells, morphine (200 μM)and lidocaine (10 μM), 12 h	AMPK-SOCS3	Zhang et al. (2017)
Atorvastatin	*in vivo*: mice, morphine (20 mg/kg, s.c., b.i.d.), 9d, atorvastatin (5, 10, 20 mg/kg, i.p., b.i.d.), 30 min before morphine administration, 9d	iNOS, TLR4, TNF-α	Pajohanfar et al. (2017)
Procyanidine	*in vivo*: mice, morphine (10 mg/kg, s.c., b.i.d.), Q12 h, 7 d, procyanidine (20, 40 or 80 mg/kg, i.g., b.i.d.)15 min before morphine administration, 7 d *in vitro*: BV2 cells, morphine (200 μM) or LPS(1 μg/mL) and procyanidine (1‰ DMSO), 12 h	p38 MAPK-NLRP3	Cai et al. (2016)

a.c.: subcutaneous injection; i. t.: intrathecal injection; i. g.: intragastric administration; i. p.: intraperitoneal injection; q. d.: *quake die* (once a day); b. i.d.: *bis in die* (twice a day); Q12 h: *Quaque 12 hora* (Once every 12 h).

### 4.2 Potential therapeutic targets

#### 4.2.1 AMPK signal

5′-adenosine monophosphate-activated protein kinase (AMPK), an AMP-dependent protein kinase, is a heterotrimeric Ser/Thr protein kinase that regulates energy homeostasis and metabolic stress by altering the cellular AMP: ATP ratio and is a key molecule in the regulation of biological energy metabolism (Zhang et al., 2009). AMPK, when activated, functions on its own primarily by inhibiting mammalian targets of rapamycin (mTOR)signalling (Melemedjian et al., 2011; Wang et al., 2024). Activation of AMPK inhibits the morphine induced activation of microglia (Han et al., 2014; Pan et al., 2016; Wan et al., 2022; Zhang et al., 2017) and suppresses neuroinflammation and reduces morphine tolerance by inhibiting MAPK signalling as well as increasing the suppressor of cytokine signalling 3(SOCS3) in microglia (Pan et al., 2016; Shirooie et al., 2020; Wan et al., 2022; Zhang et al., 2017). It has been shown that activation of AMPK promotes the conversion of microglia to the M2 type, thereby reducing neuroinflammation (Xu Y. et al., 2015). In recent studies, metformin was able to reduce morphine tolerance by activating AMPK, and some other AMPK agonists such as resveratrol/AICAR have also been shown to inhibit the development of morphine tolerance (Gabriel and Streicher, 2023; Han et al., 2014). In addition, lidocaine also indirectly activates AMPK, decreasing levels of pro-inflammatory cytokines and reducing morphine tolerance (Zhang et al., 2017). Therefore, these studies reveal the promise of AMPK as a new therapeutic target for morphine tolerance.

#### 4.2.2 Additional targets

Several other signals have been shown in recent studies to be involved in the process of morphine tolerance. The microglia-specific subtype of Ca^2+^-activated K^+^ (BK) channels is a potential therapeutic target. Paxillin, a selective inhibitor of BK channels, attenuates morphine tolerance by inhibiting BK channels in microglia. Activation of BK channels promotes the expression of P2X4Rs on the cell membrane, thereby regulating the release of BDNF (Hayashi et al., 2016). Mrg receptors are Mas-related gene receptors that belong to the G protein-coupled receptor family and are found in both humans and rodents. Previous studies have shown that the rat MrgC receptor is partially homogeneous with the human MrgX1 receptor (Dong et al., 2001). Whereas, recent studies have found that BAM8-22, an analogue of the endogenous opioid peptide BAM22, has a high affinity for the MrgC receptor and it is a highly specific agonist of the MrgC receptor. Intrathecal injection of BAM8-22 reduces morphine tolerance and enhances the analgesic effect of morphine (Zhang et al., 2019). Accordingly, the human MrgX1 receptor is expected to be a new target for treatment. In addition, we summarized potential targets that may inhibit morphine tolerance (Table 2).

**TABLE 2:** Potential targets to inhibit morphine tolerance.

Therapeutic targets	Methods	Related mechanisms	Ref.
RTP4 in the Hypothalamus	RTP4 condition knockdown	upstream: TLR4/MAPK	Fujita et al. (2022)
VGluT2	Inhibits or knockdown spinal VGluT2	upstream: BDNF/TrkB downstream: Inhibits glutamate release; Inhibits the release of inflammatory factors; Inhibits microglial activation	He et al. (2022)
TCF7L2	TCF7L2 knockdown	downstream: Inhibition of TLR4 expression; Inhibits the expression of inflammatory factors; Inhibits microglial activation	Chen et al. (2021)
Peli1	Peli1 knockdown	downstream: Inhibits K63-linked ubiquitination of TRAF6 in the spinal cord; Inhibits MAPKs signal activation; Inhibits microglial activation	(Wang L. et al., 2020)
CatS	Inhibits CatS	upstream: P2X7R downstream: CX3CL1-CXCR1 Inhibits microglial activation; Inhibits phosphorylation of p38 MAPK	Xiao et al. (2019)
Mice: MrgC receptors human: MrgX1 receptors (Dong et al., 2001)	Activate MrgC	upstream: BAM8-22 downstream: Inhibits the activation of microglia; Inhibits the expression of P2X4R; Inhibits the expression of TLR4	Zhang et al. (2019)
BK channel	Inhibits BK channel	downstream: P2X4Rs/SOCE	Hayashi et al. (2016)

RTP4, Receptor transporter protein 4; VGluT2, vesicle glutamate transporter; TCF7L2, a risk gene for schizophrenia and autism; Peli1, E3 ubiquitin ligase Pellino1; CatS, Cathepsin S; Mrg receptors, Mas-related gene receptors; BK, channel, Ca^2+^ activated K^+^ channel; SOCE, store operated calcium entry.

## 5 Summary

The molecular mechanisms of morphine tolerance are complicated and multiple, in addition to the inflammatory response caused by glial cells, some classical theories include receptor desensitisation, phosphorylation and receptor endocytosis, etc. Opioid receptor (OR) plays an important role as a target of direct action of morphine, therefore, the study of opioid receptor is also one of the crucial for the study of morphine tolerance. The current study shows that OR receptor desensitisation can lead to morphine tolerance, which may be related to the uncoupling of G proteins from OR receptors (Badshah et al., 2024). And phosphorylation of some OR targets may also contribute to tolerance, but it is still controversial (Allouche et al., 2014). Moreover, Downregulation is not necessary for tolerance (Gomes et al., 2002; Polastron et al., 1994). Whereas, MOR internalisation mitigates tolerance (Allouche et al., 2014). Indeed, there are more pathways involved, such as oxidative stress and nitric oxide pathways (Badshah et al., 2024), but these are beyond the scope of this review. We focus more on the activation associated with microglia, the signalling pathways that follow activation and the release of pro-inflammatory mediators that are triggered.

There is growing evidence that long-term morphine administration leads to tolerance and microglia activation. More research has focused on the mechanisms of morphine-induced microglial activation. P38 MAPK plays an important role in microglia activation. P38 MAPK acts as the centre of the signalling pathway within microglia and is regulated by a variety of signalling pathways. This includes upstream TLR4, phosphorylated TAK1, CGRP and P2X7R (Lehnardt et al., 2003; Olson and Miller, 2004). P38 MAPK signalling, when activated, regulates the release of cytokines such as IL-1β, IL-6 and IL-18 (Liu et al., 2019; Wang et al., 2009), further exacerbating morphine tolerance. One of its upstream signals is EGFR, and inhibition of EGFR inhibits morphine-induced activation of ERK1/2 signalling in a mouse model of cancer pain (Yang et al., 2021a; Yang et al., 2021b). In addition, JNK, a downstream signal of MOR, is able to regulate the activation of PDGFRβ, which in turn regulates the activation of microglia (Li et al., 2020). Several studies have demonstrated that the JNK signalling pathway in astrocytes regulates MOR expression and morphine tolerance (Hu et al., 2021; Sanna et al., 2020), suggesting that JNK signalling also plays an important role in tolerance. Glutamate, one of the more important excitatory neurotransmitters in the CNS, is involved in the morphine-induced intermodulation of P2X7 and P2X4 receptors. Briefly, P2X4R promotes glutamate expression through BDNF regulation of VGluT2, and increased glutamate concentrations in the cerebrospinal fluid lead to the release of excess ATP from glial cells which in turn activates P2X7R (Liu G. J. et al., 2006; Wen et al., 2004; Zhou et al., 2010). Once activated, this in turn causes the release of ATP and glutamate (Ye et al., 2003). A recent study found that P2X7R is an important signal regulating mitochondrial energy metabolism (Sarti et al., 2021). Combined with previous studies that ROS is associated with the activation of the NLRP3 inflammasome during morphine tolerance (Juliana et al., 2012), we speculate that P2X7R may activate the NLRP3 inflammasome by regulating mitochondrial production of ROS, although this speculation needs to be confirmed experimentally.

In addition to signalling pathways within microglia, actions between microglia and neurons also influence the course of morphine tolerance. Wang W et al. found that microglia, when activated, release CXCL10 and act on CXCR3 in neurons, and that minocycline or CXCR3 inhibitors were able to attenuate tolerance (Wang et al., 2017). Interestingly, chronic morphine administration causes increased expression of Monocyte chemoattractant protein (MCP-1) on neurons, and intrathecal injection of neutralizing antibodies to MCP-1 inhibits morphine-induced microglia activation and suppresses tolerance (Liu et al., 2017; Zhao et al., 2012). This suggests that there is a crosstalk between neurons and glial cells in this process. Some progress has been made regarding the mechanisms of morphine tolerance and these studies have provided new targets for delaying morphine tolerance, but further research is still needed.

In addition to tolerance, opioids have another common and troubling side effect - opioid induced hyperalgesia (OIH). Dose increases caused by tolerance can exacerbate nociceptive hypersensitivity and put patients at greater risk. Therefore, reducing morphine tolerance could prevent dose increases and more severe OIH. The exact mechanism still requires further research, and addressing this issue could help many patients suffering from pain in the clinic.

Some drugs have been used in combination with morphine with some success in animal studies, for example, some drugs for diabetic patients: metformin, glibenclamide, the lipid-lowering drug atorvastatin, and the hypertensive drug candesartan (Table 1). In clinical practice, neuralgia in diabetic patients is notorious, and most often the analgesic drugs are ineffective. Therefore, it is still unknown whether these drugs can play a role in reducing morphine analgesic tolerance in the clinic, and more experiments are needed to see whether these drugs can be promoted for use in combination with other opioids. What is certain, however, is that the development of drugs that can be used in combination with opioids for these therapeutic targets is one of the future directions. In addition, researchers have found that endomorphin analogues can produce the same antinociceptive sensations as morphine without activating glial cells (Zadina et al., 2016; Zhang et al., 2019). This suggests that similar alternative drugs that have the same analgesic effect without side effects or with minimal side effects are also one of the future directions for opioid analgesics.

## 6 Conclusion

**FIGURE 3:**
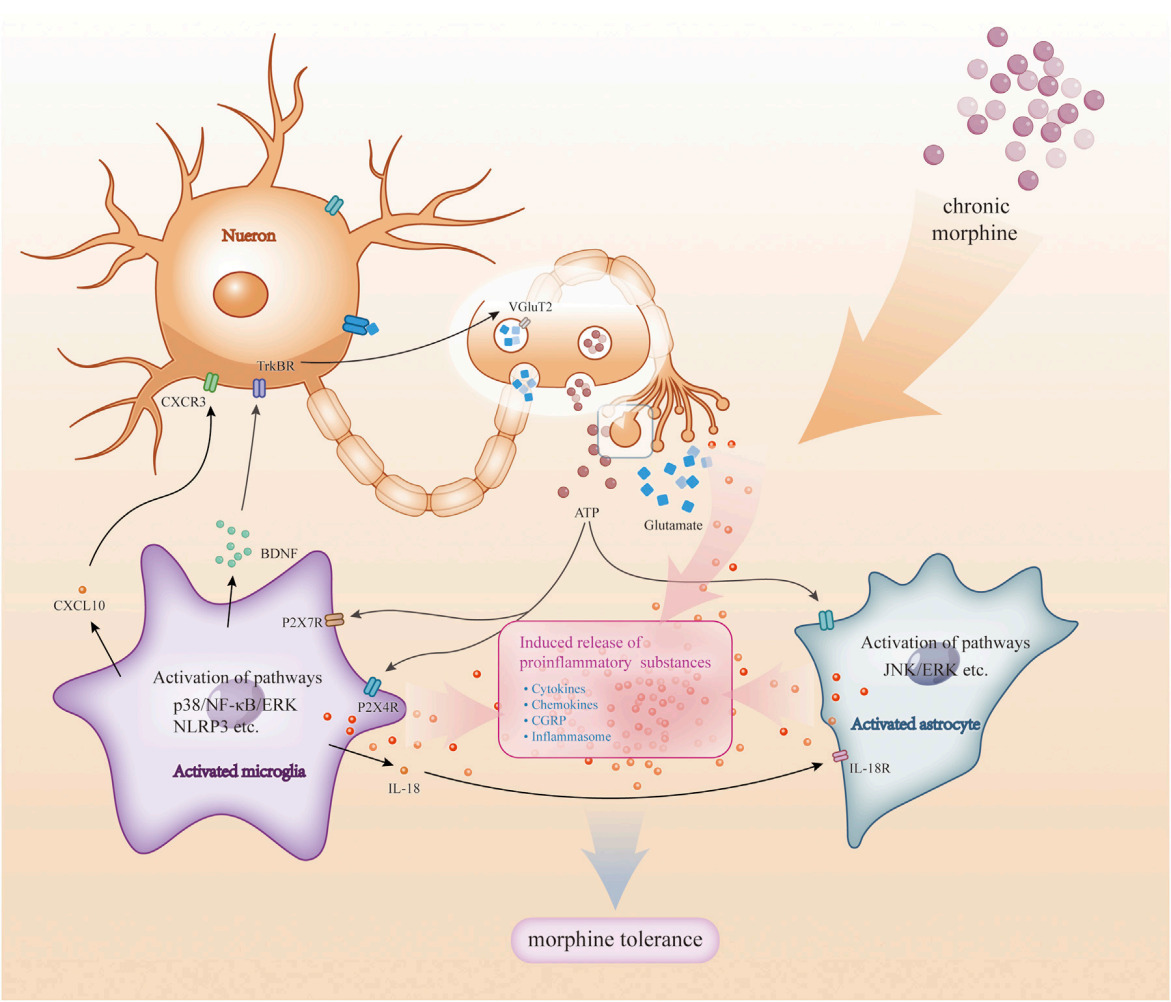
Crosstalk among microglia and astrocytes with neurons. During chronic morphine administration, there is crosstalk between neurons and glial cells (e.g., astrocytes, microglia). Substances such as ATP and glutamate released by neurons stimulate glial cell activation: activation of microglia activates signalling pathways (e.g., p38, NF-kB, ERK, etc.) and triggers the release of pro-inflammatory substances, which in turn act on neurons and glial cells; also, when astrocytes are activated, the activation of signalling pathways (e.g., JNK, ERK, etc.) also triggers the release of pro-inflammatory mediators, which together act on glial cells and neurons. These pro-inflammatory substances further exacerbate this response, leading to more glial cell activation and inflammatory response, and ultimately morphine tolerance.

This review details the links between morphine tolerance and microglia, including microglia activation and specific signalling pathways. We suggest that the mechanisms involved in morphine tolerance are complex, with crosstalk between neurons and glial cells as well as between different glial cells (Figure 3). Therefore, it is equally important to study neurons with astrocytes and oligodendrocytes. Currently, relevant studies have focused on animal experiments, and more clinical studies will likely be needed in the future to address this issue. In addition, this review summarises the drugs that have achieved success in animal studies so far, and these results provide directions for future research.
